# Mutations in the anti-sigma H factor RshA confer resistance to econazole and clotrimazole in *Mycobacterium smegmatis*

**DOI:** 10.1099/acmi.0.000070

**Published:** 2019-10-29

**Authors:** Héctor R. Morbidoni, Agustina I. de la Iglesia, Virginia Figueroa, Cecilia Di Capua, Thomas R. Ioerger, Tanya Parish

**Affiliations:** ^1^​ Laboratorio de Microbiología Molecular, Facultad de Ciencias Médicas, Universidad Nacional de Rosario, Rosario, Argentina; ^2^​ Department of Computer Science and Engineering, Texas A&M University, College Station, TX, USA; ^3^​ TB Discovery Research, Infectious Disease Research Institute, Seattle, WA, USA; ^†^​ Present address: Instituto de Biología y Medicina Experimental (IBYME), CONICET, Buenos Aires, Argentina

**Keywords:** *Mycobacterium smegmatis*, *Mycobacterium tuberculosis*, econazole, RshA

## Abstract

Azole drugs such as econazole, are active on *Mycobacterium tuberculosis* and *Mycobacterium smegmatis *; however, the identification of their target(s) is still pending. It has been reported that mutations in the non-essential system *mmp*L5-*mmp*S5 conferred resistance to econazole in *M. tuberculosis *. We herein report that an azole-resistant mutant screen in *M. smegmatis* rendered mutations in *rsh*A, encoding a non-essential anti-sigma H protein.

*Mycobacterium tuberculosis *, the causative agent of tuberculosis, is still one of the three major infectious agents leading to death of more than 1.5 million people and 10 million new cases per year [1]. The treatment of tuberculosis is lengthy and cumbersome, requiring from six to 24 months, and is currently jeopardized by the rise of drug-resistant strains that are refractory to most if not all the known available drugs [2]. Those facts underscore the need for novel therapeutic strategies; however in the last 10 years few additions to the therapeutic portfolio have been made [3].

The polycyclic azoles are Cytochrome (Cyt) P450 inhibitors, which have been used in the treatment of fungal pathogens, on which they are active by inhibition of Cytochrome P450 (Cyp)51, an essential lanosterol 14α–demethylase, involved in the synthesis of the cell membrane component ergosterol [4]. Interestingly, the members of the genus *Mycobacterium* contains a large array of Cyps, while the *M. tuberculosis* genome encodes 20 Cyps, *Mycobacterium bovis* has 18 and *M. smegmatis* has 39 of them [5, 6]. The essentiality of several of these genes was examined due to the interest Cyps represent as drug targets [7, 8]. Importantly activity of azoles on *M. tuberculosis* (*in vitro*, *ex vivo* and in a murine model) and *M. smegmatis* has been described [9–12], fostering the attention to the identification of their targets. Of note, mycobacteria does not contain ergosterol in their cell-membrane composition and do not synthesize sterols [13, 14], pointing to a novel mechanism of action of azole drugs in these bacteria different to that taking place in fungi. Disappointingly, transposon saturation mutagenesis technologies have shown that only *cyp*136 (encoded by Rv3059) confers an *in vitro* growth advantage while none of the remaining members of the Cyp mycobacterial repertoire was essential [15]. More puzzlingly, deletion of *cyp*125, although not essential, caused a tenfold increase in the susceptibility of *M. tuberculosis* to econazole (Eco) [16]; thus none of the CYPs fulfilled the criteria for being considered a target for azoles, although there is a large body of information on binding of those drugs to CYPs [17–20]. Moreover, we have previously demonstrated that imidazoles are bactericidal to *M. tuberculosis *, and that they generate a large increase in reactive oxygen species (ROS) [16]. However, that event is not related to the mechanism of killing of azoles since over-expression of *sod*C or *kat*G, enzymes detoxifying oxygen radicals in *M. tuberculosis *, had no effect on the susceptibility to those compounds. Adding complexity to the scenario, it was also found that Eco treatment of wild-type and an Eco-resistant *M. tuberculosis* mutant brought about comparable changes in carbohydrates, amino acids and energy metabolism suggesting that stress adaptation may play a role in the resistance to this compound [16]. Until now, only one mechanism of resistance has been genetically demonstrated in *M. tuberculosis *, consisting in the up-regulation of the efflux pump system encoded by *mmp*S5–*mmp*L5 genes due to mutations either in the Rv0678 gene, involved in the transcriptional regulation of this efflux system, or in its putative promoter/operator region [21]. In a recent review, Briffotaux *et al.* analysed the role of MmpL5-MmpS5 as an efflux pump in *Mycobacterium* species [22]. MmpL5-MmpS5 are present in all mycobacterial species except for *M. leprae *, differing in the nature of the transcriptional regulator; while *M. tuberculosis* and other slow growers contain a MarR transcriptional regulator, fast-growing species such *as M. smegmatis* have a transcriptional regulator belonging to the TetR family [22]. The fact that it was demonstrated that Rv0678 binds ‘*in vitro*’ not only the *mmp*L5-*mmp*S5 promoter but also those of *mmp*L4-*mmp*L4 and *mmp*L2-*mmp*S2 raises a question on other effects that this mechanism of Eco resistance may have in *M. tuberculosis* [22]. So far, in spite of the identification of the MmpL5-MmpS5 system as the only genes reported as related to resistance to azole drugs in *M. tuberculosis *, the identity of the target(s) for those drugs in this pathogen remains elusive.

We hypothesized that the use of *M. smegmatis *, a saprophytic species with an ample range of growth temperatures that allows for the isolation of temperature-sensitive (Ts) mutants – thus indicative of mutations causing loss of function in essential genes – would allow for the identification of a candidate target for azole drugs. A similar strategy was successfully carried out by Vilchéze *et al*., leading to the identification of InhA – target for the first-line anti-tubercular drug isoniazid – in *M. smegmatis* [23]. To that aim, we first examined the influence of medium composition on econazole activity. The reports on MIC_99_ values for Eco and clotrimazole (Clo) on *M. smegmatis* widely vary depending on the culture medium and methodology used and are specially low in complex medium such as LB (MIC_99_=2 µg ml^−1^ and 0.5 µg ml^−1^, respectively [10]) when compared to chemically defined medium such as Sauton medium (MIC_99_=20 µg ml^−1^ and 15 µg ml^−1^, respectively [10]. Eco and Clo inhibit *M. tuberculosis* on solid Middlebrook 7H11ADS glycerol agar medium (MIC_99_=8 µg ml^−1^ in both cases [21]) or Middlebrook 7H9 ADS glycerol (7H9 ADS Gly for short) broth (MIC_99_=15 µg ml^−1^ and 17 µg ml^−1^, respectively [24]. Thus, in order to choose a growth medium for our screening, we analysed the influence of its composition on azole activity on *M. smegmatis *. In our hands Eco and Clo inhibited *M. smegmatis* with comparable efficacy on Middlebrook 7H9 ADS Gly agar plates (MIC_99_=15 µg ml^−1^ and 12 µg ml^−1^, respectively) and Mueller–Hinton agar plates (MIC_99_=1.0 µg ml^−1^ and 0.5 µg ml^−1^). Interestingly, while assessing the influence that the different components of the growth medium could have on Eco activity, we found that the addition of dextrose (0.4 % w/v) resulted in a twofold increase in the MIC_99_Eco in Mueller–Hinton agar and LB agar media for the wild-type strain *M. smegmatis* mc^2^155. Conversely, using a reconstitution approach to a ‘basal’ Middlebrook 7H9 ClNa agar medium, we noticed that Eco was much more active when 0.5 % (w/v) glycerol was the carbon source (MIC_99_=2 µg ml^−1^); this value did not change upon addition of other components present in LB [0.2 % (w/v) yeast extract or 0.1 % (w/v) casaminoacids]. In contrast, the presence of 0.4 % (w/v) dextrose as the carbon source increased resistance to Eco (MIC_99_ Eco=10 µg ml^−1^) yielding small size colonies but in comparable numbers to those on control plates up to that concentration. Since the reported MIC for *M. tuberculosis* when Middlebrook 7H11 ADS-Gly (thus, containing both dextrose and glycerol), Sauton or Youngman liquid media (both containing only glycerol as the carbon source) are used ranges from 8 to 20 µg ml^−1^ as cited above, we hypothesized that other components present in those three growth media could also be influencing the activity of the drug; thus we tested Zn^2+^ (10–50 µg ml^−1^) addition of which consistently helped *M. smegmatis* to grow in Mueller–Hinton agar and LB agar containing Eco 5 µg ml^−1^; however growth was again comparable to control plates in colony numbers but smaller in size especially at the higher concentration. The addition of this cation to Middlebrook 7H9 dextrose caused only a marginal but reproducible increase in the MIC (MIC_99_ Eco=12–15 µg ml^−1^). The activity of Clo was equally affected by growth medium composition (data not shown). Taken as a whole, our findings explain the reported differences on azole activity on mycobacteria as the nature of the growth medium clearly influence the susceptibility to those drugs.

On the rationale that the ability of *M. smegmatis* to grow at a wide range of temperatures would allow obtaining temperature-sensitive (Ts) mutants affected in essential pathways linked to resistance to azoles, we next set to isolate Ts Eco- and Clo-resistant mutants in *M. smegmatis* [using each time five independent cultures grown at 30 °C from a small inoculum ≈10^5^ c.f.u. ml^−1^ by plating aliquots (10^7^–10^9^ c.f.u.) on 7H9ADSGly agar plates (chosen in order to be comparable to previously reported *M. tuberculosis* mutant-selection medium [21]) containing 50 µg ml^−1^ of Eco or Clo]; however this strategy failed as none of the obtained azole-resistant mutants displayed a Ts phenotype in spite of several attempts. Notwithstanding this, we obtained several *M. smegmatis* non-Ts mutants resistant to Eco and Clo (frequency of isolation ~4×10^−8^). MIC values for those mutants were of two–fivefold MIC (30–75 µg ml^−1^) for Eco and two–threefold MIC (30–40 µg ml^−1^) for Clo. A set of four mutants was further characterized on the basis of phenotypic differences such as colony morphology and sliding motility (indicative of cell-wall alterations), generation time and level of resistance to azoles. While the morphology of the colonies and duplication times of mutants Eco-R1, Eco-R2 and Eco-R8 were comparable to those of the parental strain; mutant Eco-R6 showed a much longer duplication time (4.6 h) (Fig. 1). Interestingly, mutants differed in their MIC Eco in MH agar plates, while mutants Eco-R1 and Eco-R2 were resistant to 4 µg ml^−1^, mutants Eco-R6 and Eco-R8 were resistant to 16 µg ml^−1^ and 32 µg ml^−1^, respectively. Although mutant Eco-R2 displayed a smooth colony morphology and higher Congo red binding than the other mutants, we hypothesized that mutations increasing the dispensable synthesis of glycopeptidolipids (known to be inhibited by azoles [10]) would explain that morphotype.

**Fig. 1.**
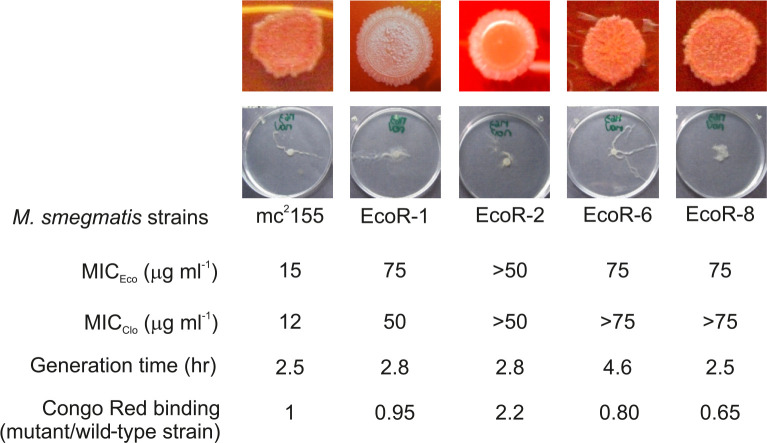
Characterization of *M. smegmatis* Eco-resistant mutants. Upper row: the parental strain *M. smegmatis* mc^2^155 and its derived spontaneous Eco-resistant mutants EcoR1, EcoR2, EcoR6 and EcoR8 were grown on 7H9-ADS-Gly agar plates supplemented with Congo Red dye (100 µg ml^−1^) for 3 days at 37 °C; colonies were observed visually and photographed with a Zeiss-Stemi 2000 lens at x2 magnification. Lower row: sliding motility of those strains was tested by applying a 10 µl aliquot of each culture onto semi-solid M63-based medium and monitored after 7 days of incubation at 37 °C. Finger-like extensions appeared and spread outwards from the central inoculation point. The resistance to Eco and Clo of each strain was determined by plating aliquots containing ≈ 10^3^ c.f.u. on plates of 7H9-GlyADS medium containing increasing concentrations of each compound and visually counting colonies after 5 days of growth at 37 °C. MIC_99_ was considered the drug concentration that reduced 99 % the c.f.u. counts when compared to the plates with no drug. Generation times for each strain were calculated from growth curves performed in 7H9ADS- Gly - 0.5 % Tw. Each experiment was done in triplicate. Binding of the dye Congo Red was carried out as described previously [33]. Briefly, cultures of each strain were grown at 37 °C in medium supplemented with Congo Red dye (100 µg ml^−1^) for 3 days. Cells were collected and washed, and the washed pellets were resuspended in acetone and Abs488 of Congo Red measured. Relative binding was calculated as OD_488_ pellet/dry weight for each mutant relative to the OD_488_ pellet/dry weight of the parental strain.

As mentioned above, Milano *et al*. reported that isolated spontaneous *M. tuberculosis* and *M. bovis* BCG mutants resistant to azoles had mutations in the transcriptional regulator Rv0678, which led to increased transcription of the Rv0677c and Rv0676c genes corresponding to the proteins MmpS5 and MmpL5, which conform a hypothetical efflux system belonging to the resistance–nodulation–division (RND) family of transporters [21]; those proteins have also been shown to play a role in resistance to bedaquiline and clofazimine in *M. tuberculosis* [25]. Of note, the efflux pump inhibitors (EPIs) reserpine and verapamil were able to increase the susceptibility to those drugs in wild-type and resistant mutants at low concentrations [25]. Thus, we determined the effect of the addition of sub-inhibitory concentrations of reserpine (12 µg ml^−1^) or verapamil (40 µg ml^−1^) on the activity of Eco and Clo in the parental *M. smegmatis* strain; our results showed that the addition of those EPIs failed to allow for growth in the presence of Eco at 37 °C (Fig. 2); comparable results were observed when Clo was used (data not shown); however *M. smegmatis* EcoR mutants 1, 2, 6 and 8 were able to grow in the presence of Eco at 50 µg ml^−1^ even when reserpine and verapamil were added, indicating a lack of effect of both EPIs on the resistance phenotype of those mutants (Fig. 2, only mutant Eco-R8 is shown by the sake of simplicity). Susceptibility of the mutants to several compounds (isoniazid, ethionamide, erythromycin, tetracycline, novobiocin and streptomycin) was comparable to that of the wild-type strain, while only chloramphenicol revealed differences in susceptibility with an eightfold increase in the mutants vs the parental strain (MIC=64 µg ml^−1^ and 8 µg ml^−1^, respectively, data not shown).

**Fig. 2.**
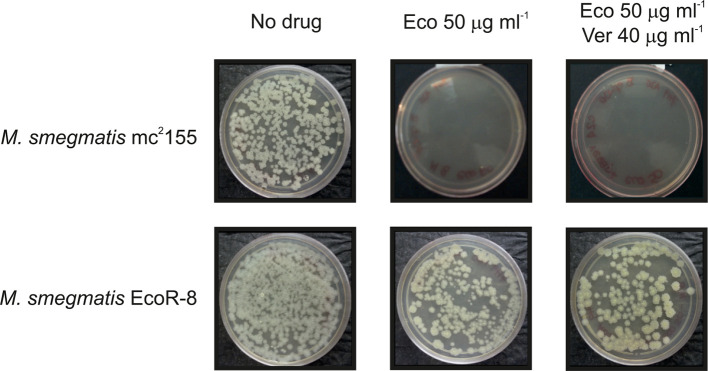
Presence of efflux-pump inhibitors, verapamil and reserpine, does not decrease the level of resistance to Eco in the *M. smegmatis* EcoR mutants. Aliquots containing 300–500 c.f.u. of the parental strain and each of the EcoR-resistant mutants were seeded into 7H9-ADS-Gly plates in the absence of drugs, in the presence of Eco (50 µg ml^−1^) or Clo (50 µg ml^−1^) or in the presence of Eco or Clo (50 µg ml^−1^ in each case) with the addition of verapamil (40 mg ml^−1^) or reserpine (12 µg ml^−1^). Counting of colonies in each condition was performed upon incubation of the plates at 37 °C for 3 days. For the sake of simplicity the results of only one mutant (EcoR 8) and one efflux-pump inhibitor (verapamil) are shown as all the strains behaved similarly regardless of which efflux-pump inhibitor was used.

We next set to determine the presence of mutations affecting the expression of the *mmp*L5-*mmp*S5 operon as described for *M. tuberculosis* by Milano *et al.* [21]. PCR amplification and DNA sequencing of the products of the *mmp*L5-*mmp*S5 chromosomal region in the four mutants failed to reveal changes in the sequence of the promoter region of *mmp*S5 (MSMEG_0227) or the intergenic region of MSMEG_0226 compared to the parental strain (data not shown).

In order to identify the mutated gene responsible for the azole resistance phenotype, we performed whole-genome sequencing of the wild-type strain (as control) and mutants Eco-R6 – having a generation time almost twice that of the parental strain – and Eco-R8 – having a generation time comparable to that of the parental strain – (Fig. 1). Chromosomal extraction, library construction and sequencing was conducting as described previously using an Illumina HiSeq 2500 and 51×51 bp paired-end reads [26]. Variants including SNPs, insertions and deletions, were identified by alignment with the *M. smegmatis* mc^2^ 155 reference sequence (GenBank accession number NC_018289.1). Analysis of the sequences obtained allowed for the identification of mutations lying on gene MSMEG_1915 (*rsh*A) in both mutants; while mutant 6 contained a T insertion at bp 246 – thus leading to a frameshift in the coding sequence and generating a shorter protein (83 residues) due to a stop codon created at bp 250 – mutant 8 had a C55Y change at bp position 174. Of note, *rsh*A, encoding an anti-sigma H protein, is present in other mycobacteria including *M. tuberculosis *, *M. bovis *, *M. leprae* and *M. abscessus* (Fig. 3a). The possible loss of function of a shortened RshA in mutant EcoR-6 or the loss of a critical C residue (C55) in mutant EcoR8 could impair RshA function and in the case of the latter mutant, may explain the differences in generation times when compared to the parental strain. No other relevant mutations were detected upon bioinformatics analysis of each sequenced genome.

**Fig. 3.**
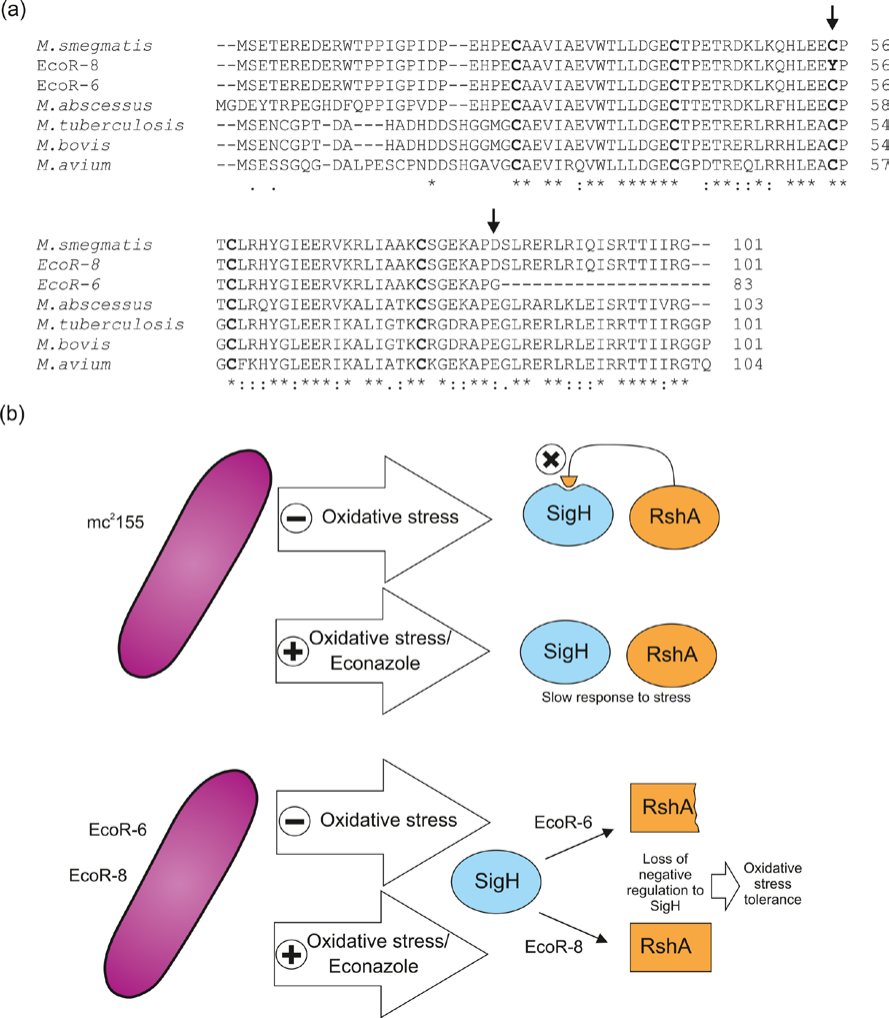
*
M. smegmatis* EcoR mutants 6 and 8 contain mutations in the anti-sigma H protein encoded by gene MSMEG_1915 (*rsh*A). (a) An aligment of RshA proteins present in mycobacteria is shown; the conserved Cys residues are displayed in bold type and the mutations detected in the EcoR6 and EcoR8 mutants are shown by arrows. (b) A model proposing the effect of the loss of RshA in the resistance to Eco in *M. smegmatis* is shown. Full sized wild-type RshA is shown as an oval, EcoR 6 RshA and EcoR8 RshA are shown as a shortened square and a full size square to depict the possible changes in SigH- RshA interactions. Materials: all chemicals used were purchased pure, from commercially available sources such as Sigma Aldrich, New England Biolabs or other vendors.

The alternate sigma factor SigH of *M. tuberculosis* is expressed under heat/oxidative stress and acts as a major regulator of several genes, including other sigma factors and redox systems [27, 28]. The SigH-dependent activation of this ample stress response system is critical for *M. tuberculosis* virulence, as it has been shown that a *sig*H mutant is highly attenuated in the mouse model of infection [29]. SigH is transcriptionally auto-regulated by its own promoter although at the post-translational level a second mechanism of regulation is through its cognate protein, RshA. This protein located 3′ of *sig*H– provides a direct mechanism for sensing oxidative stress. Both RshA and SigH are phosphorylated *in vitro* and *in vivo* by the serine-threonine protein kinase PknB; importantly phosphorylation of RshA takes place at a T94 residue in *M. tuberculosis* [27], residue that is lost in the truncated version of RshA present in mutant EcoR6. The binding of RshA to SigH is redox-dependent, constituting the manner by which SigH activity is regulated in response to oxidative stress [28] (Fig. 3b). Kumar *et al.* showed that RshA and SigH interact through salt bridges; with four Cys residues (out of the five conserved Cys present in the protein, shown in bold in Fig. 3a) playing an important role in the conformation of RshA through disulfide bond formation necessary for the coordination to a [Fe-S] cluster [30]. Importantly, Park *et al.* demonstrated that amino acid substitution of two of the cysteine residues present in RshA (C55 and C58) abrogated the formation of the complex between RshA and SigH [28]; this gives support to our identification of a C55Y mutation in mutant Eco-R8, which may alter that disulfide bond formation and hence, the complex formation. These authors have also suggested that the mechanism of phosphorylation-mediated interaction between RshA and SigH allows for a graded response to oxidative stress [28], thus loss of the reside at which phosphorylation takes place in RshA would most likely allow for maximum expression of SigH, which is convenient for the handling of the cellular oxidative stress caused by azoles. Under the light of that information both of our RshA mutants will fit into a model of resistance to azoles in which the *M. smegmatis* cell shapes its intracellular metabolism to face oxidative stress, maximizing the activity of SigH through the loss of the phosphorylation site of its cognate regulator RshA or through mutations close to the [Fe-S] cluster required for RshA conformational stability and activity. Finally, it is also relevant to indicate that it was previously reported that RshA is a weak binder of Zn^2+^ [30], which may explain our findings on the mild but noticeable protective effect of this cation in the MIC Eco of *M. smegmatis *.

Our previous work revealed that Eco treatment of *M. tuberculosis* displayed very similar changes to an untreated Eco-resistant strain, with higher levels of glucose-6-phosphate, fructose-6-phosphate, glucosamine-6-phosphate and mannose-6-phosphate as well as lower levels of N-acetylglucosamine, mannose, mannose-1-phosphate, inositol-1-phosphate and trehalose-6-phosphate [24]. Econazole treatment led to changes in the levels of glucose-6-phosphate, fructose-6-phosphate and glucosamine-6-phosphate decreased in the resistant mutant strain with econazole treatment as opposed to the increased levels observed in the wild-type strain, underscoring a complex effect of azole drugs on the mycobacterial central metabolism [24]. Other possible explanations for the effect of this micronutrient in the frame of our findings is that it is required for enzymatic function of enzymes such as fructose-1,6-bis-phosphate aldolase or Cu Zn SOD [31]. Thus, although speculative, it is possible that the mild protective effect of Zn^2+^ could be related to the activity of one or more enzymes of central mycobacterial pathways. Thus, although beyond the scope of the present work, research on this area is warranted.

In sum, our results report for the first time the identification of RshA as a novel player in resistance to azoles in mycobacteria. Importantly, this is also the first report linking mutations in *rsh*A to resistance to an antibiotic in *M. smegmatis *. It also raises the question of whether comparable mutations in the *M. tuberculosis* genome may happen and contribute to drug resistance to certain drugs, a point to address in HIV- Tb patients undergoing fungal infections. Of note, a very recent report has shown that mutations in *Mycobacterium abscessus* MAB_3542 c, encoding a RshA-like protein may be responsible for resistance to tigecycline [32], suggesting that our identification of RshA as a player in the mycobacterial resistance to Eco may not be specific to azoles but related in a general way to resistance to other antibiotics, an issue that should be addressed in the future.

So far, only mutations in Rv0678 and MSMEG_1915 have been identified mycobacteria as genes linked to azole resistance but surprisingly there is no report on other genes involved in the mechanism of action of those compounds. The failure in identifying a target for azole drugs using a Ts screen in *M. smegmatis* agrees with previous attempts to identify a target for those drugs in *M. tuberculosis* suggesting that they may not inhibit a defined target enzyme. However, our findings would allow the construction of a *rsh*A merodiploid *M. smegmatis* strain that will be valuable for the selection of new azole-resistant mutants affected in other novel genes.
